# Complete Genome Sequence of *Spiroplasma kunkelii* Strain CR2-3x, Causal Agent of Corn Stunt Disease in *Zea mays* L.

**DOI:** 10.1128/genomeA.01216-15

**Published:** 2015-10-22

**Authors:** Robert E. Davis, Jonathan Shao, Ellen L. Dally, Yan Zhao, Gail E. Gasparich, Brady J. Gaynor, John C. Athey, Nigel A. Harrison, Nicole Donofrio

**Affiliations:** a Molecular Plant Pathology Laboratory, USDA-Agricultural Research Service, Beltsville, Maryland, USA; b Department of Biological Sciences, Towson University, Towson, Maryland, USA; c Ft. Lauderdale Research & Education Center, University of Florida, Davie, Florida, USA; d Department of Plant and Soil Sciences, University of Delaware, Newark, Delaware, USA

## Abstract

*Spiroplasma kunkelii* causes corn stunt disease of *Zea mays* L. in the Americas. Here, we report the nucleotide sequence of the 1,463,926-bp circular chromosome and four plasmids of strain CR2-3x. This information will facilitate studies of *Spiroplasma* pathogenicity and evolutionary adaptations to transkingdom parasitism in plants and insect vectors.

## GENOME ANNOUNCEMENT

Spiroplasmas are characteristically helical, motile cell-wall–less prokaryotes (1, 2). While the first recognized spiroplasma was discovered in a diseased plant and insect vector (1–3), non-plant-pathogenic spiroplasmas have been reported as residents on surfaces of flowers, as symbionts of ticks and diverse insect species, and as pathogens of honey bee, crustaceans, and, apparently, humans (4–11). Recently, a human-infecting spiroplasma was identified as a strain of the horse fly (*Haematopota* sp.) symbiont, *Spiroplasma turonicum* (11). These discoveries underscore a prediction made almost 40 years ago, that spiroplasmas in their widely diverse habitats exhibit a constant link with arthropods (4).

Here, we report the complete nucleotide sequence of the circular chromosome and four plasmids of *Spiroplasma kunkelii* strain CR2-3x, which was isolated in axenic culture from naturally infected, field-grown plants of corn (*Zea mays* L.) in Costa Rica (12). Genomic DNA was extracted from a culture of *S. kunkelii* CR2-3x grown in liquid medium as previously described (12). Nucleotide sequencing was carried out using the NGS platform Pacific Biosciences (PacBio) single-molecule, real-time sequencing system, in which 29,904 reads were obtained, totaling 174,151,004 nucleotides. The *N*_50_ read length was 9,508 nucleotides; the mean read length was 5,823 nucleotides; and the average reference consensus concordance was 100.00%. The assembled, circular chromosome of 1,463,926 bp has an overall base composition of 24.97 mol% G+C; the average coverage per base position was 97.35×. The four plasmids were 22,558, 14,615, 20,501, and 7,576 bp in size and had base compositions of 27.64, 28.14, 24.20, and 21.50 mol% G+C, respectively. The assembled chromosome and plasmids were put through GeneMark.hmm (13) annotation and were curated by manual inspection using Artemis (14) as an annotation platform. The programs tRNAscan-SE version 1.21 and RNAmmer (15) were used to predict regions encoding tRNAs and rRNAs. The chromosome has 1,646 protein coding regions (CDSs), multiple insertions of spiroplasma virus sequences, one set of rRNA genes, and 33 tRNA genes.

Phylogenetically, *S. kunkelii* clusters with other wall-less bacteria in the class *Mollicutes* and is most closely related to the plant pathogens *S. citri* and *S. phoenicium*, and to the honey bee pathogen *S. melliferum* (16–18). Availability of the *S. kunkelii* complete genome should facilitate studies to elucidate the evolutionary biology of plant-pathogenic spiroplasmas.

### Nucleotide sequence accession numbers.

This genome project has been deposited in GenBank under the Bio-Project ID PRJNA270865, BioSample accession number SAMN03269975: *Spiroplasma kunkelii* CR2-3x (Taxid 47834), and accession numbers CP010899 (chromosome), CP012423 (plasmid pSKU226), CP012424 (plasmid pSKU205), AY528560 (plasmid pSKU146, NC_006400), and CP012425 (plasmid pSKU76). The sequence versions described in this paper are versions CP010899.1, CP012423.1, CP012424.1, AY528560.1 (NC_006400.1), and CP012425.1.
